# Capturing the Complexity of Trauma Care: A Data Infrastructure for a Learning Healthcare System in Traumatology

**DOI:** 10.3390/jcm15218571

**Published:** 2026-11-03

**Authors:** Frederique J. C. van Eerten, Steven Ferree, Jan-Hendrik Venhuizen, Mark C. H. de Groot, Saskia Haitjema, Falco Hietbrink

**Affiliations:** 1 Department of Trauma Surgery, University Medical Center Utrecht, 3508 GA Utrecht, The Netherlands; 2 Central Diagnostic Laboratory, University Medical Center Utrecht, Utrecht University, 3508 GA Utrecht, The Netherlands

**Keywords:** trauma care, Learning Healthcare System, trauma research, data integration, quality improvement

## Abstract

**Background:** Trauma care is inherently complex due to wide variability in patients, injury patterns, possible treatment strategies, and achievable outcomes that dynamically interact and influence the risk of complications and long-term functional recovery. Mortality-focused research insufficiently reflects this complexity and the evolving goals of trauma care. A Learning Healthcare System offers an opportunity to learn from routine clinical care by integrating data and translating findings into feedback. **Objective:** This paper proposes a comprehensive framework for continuous evaluation and improvement of trauma care that integrates patient and injury characteristics with care processes, complications, and outcomes beyond mortality, and describes an institutional data infrastructure that can support this framework. **Approach:** A conceptual framework based on routine care data is described, together with an institutional example combining the Dutch National Trauma Registry, the Utrecht Patient-Oriented Database, and a specialty complication registry. No new patient-level data were created or analyzed, and the effectiveness of the integrated infrastructure was not evaluated. Maintaining these databases requires dedicated professionals, data managers, structured documentation, multidisciplinary collaboration, and continuous quality control. **Conclusions:** Linking complementary registries provides a foundation for a Learning Healthcare System in complex trauma care. Combining established mortality and morbidity reviews with the opportunities offered by large, integrated databases may facilitate the implementation of a Learning Healthcare System in trauma care.

## 1. Introduction

Trauma patients account for a substantial proportion of all hospital admissions worldwide and contribute significantly to hospital mortality and comorbidity [1]. Over recent decades, trauma care has improved markedly, partly due to the implementation of inclusive trauma systems in multiple countries [2]. These systems are designed to optimize usage of available capacity and correct allocation of resources. As such, these systems are characterized by regional collaboration, structured levels of trauma care facilities and a continuum of care [2,3,4]. Within this model, trauma is acknowledged as a surgical disease, requiring a holistic perspective on patient management [3].

Treating trauma as a disease means treating the patient as a whole, rather than a collection of isolated injuries. Effective and patient-tailored decision-making requires integration of pre-existing comorbidities, physiology, immunology, visceral injury management, and extremity/fracture care [5]. As a result, trauma care is inherently complex.

This complexity necessitates continuous evaluation of the quality of care and targeted improvement where possible. Capturing the full scope of trauma care in quality analyses requires a comprehensive approach. Outcome analyses should therefore encompass the entire continuum of care, including clinical decision-making, medical and invasive interventions, logistical processes, and principles of personalized medicine. However, although current trauma research often focuses primarily on crude and adjusted mortality as it is the most robust outcome, it insufficiently reflects a more in-depth perspective [6].

This limitation is particularly relevant given the characteristics of the trauma population and recent developments in trauma care. Severely injured trauma patients are often a healthy and relatively young patient group, who acutely suffer from a disease (the injuries), and often remain limited in their functional capabilities afterwards [7]. Furthermore, mortality rates have been reduced drastically and have reached a low-level plateau in the past years in the Netherlands [2]. The high impact of functional disability on these often young patients, combined with saturated mortality rates, suggests the need for analyses of additional outcomes such as complication rates, failure to rescue and functional outcomes when evaluating trauma care [8,9].

To continuously evaluate and improve such a complex care pathway, a Learning Healthcare System provides a promising framework [10,11]. In a Learning Healthcare System, routinely collected clinical data are systematically integrated, analyzed, and translated into actionable feedback for healthcare professionals, thereby creating a continuous cycle of learning and quality improvement [10,11,12]. This approach has already proven valuable in cardiovascular care [12]. Structured registration of clinical data and continuous monitoring have improved guideline adherence, reduced missing risk factors, and enabled evaluation of the complete care continuum [10,11,12]. Similar principles could be applied to trauma care, where integrating patient characteristics, injury patterns, treatment decisions, complications, and outcomes would facilitate continuous learning and support data-driven improvements in clinical practice.

Existing trauma quality-improvement initiatives also provide an important context. Programs such as the American College of Surgeons Trauma Quality Improvement Program (ACS TQIP) and Dutch National Trauma Registry (DNTR) use registry data for benchmarking and feedback. The contribution of the present framework is the combination of standardized national injury data with detailed hospital-based physiological, laboratory, treatment, and complication data. This allows more in-depth analysis at the institutional level than is possible with national trauma registry data alone. The present framework should therefore be considered complementary to existing registry-based quality-improvement systems [13,14].

The complexity of trauma care demands a holistic and multidisciplinary approach, which should also be reflected in research [2]. This paper aims to illustrate the value of combining complementary routine care data sources to capture patient and injury characteristics, care processes, complications, and (functional) outcomes beyond mortality. It presents a conceptual framework for an integrated data infrastructure that can provide the foundation for a Learning Healthcare System, rather than a research protocol or an effectiveness study.

## 2. Why Evaluating Trauma Care Is Complex

As stated above, trauma care is challenging because the high variability in patients, injuries and care all influence the divergence in outcomes. As a result, evaluating trauma care is complex. Understanding these contributors of variability is essential for designing comprehensive analyses.

### 2.1. Variability in Patients and Injuries

Patient-specific aspects that might affect outcomes include age, gender, body composition and comorbidities [15,16,17]. In addition, variation in injury mechanism and severity impacts the burden of trauma and as such outcome as well. Next, the subsequent impact of injury and systemic responses to it vary across injuries to different body regions and are unique to each patient [18,19].

### 2.2. Variability in Care and Timing

On a level of care provision, treatment strategies in terms of timing and extent of procedures affect outcomes. Surgically, the two ends of the strategy spectrum are Damage Control Surgery (DCS) and Early Total Care (ETC). DCS consists of staged, shorter interventions to stabilize patients and prevent systemic complications. On the other hand, ETC performs definitive interventions early, reducing local complications and aims for early functional recovery [20]. In current practice, Early Appropriate Care (EAC) is applied as a dynamic mix of both strategies: interventions and their extent are timed based on continuous evaluation of the patient’s systemic state. Finally, non-surgical factors, such as (preventative) medication or ICU admission, ventilator strategy and resuscitation protocols all affect the outcome and risk of complications.

### 2.3. Variability in Outcomes

Outcomes include crude clinical measures such as mortality and length of stay, but also complications and functional recovery [8,9]. Functional outcomes are frequently more subjective and are influenced by the patient’s pre-injury state and the expectations of both patient and physician [21]. The Olympic Model brings together injury-specific objective outcomes, general subjective functioning, patient expectations, and physician technical judgment [22]. These domains distinguish technical recovery from the patient’s experience of recovery and whether expectations are met. Including complications and patient-reported outcomes is crucial for a holistic view of trauma care [22,23].

## 3. A Proposed Framework for Comprehensive Evaluation of Trauma Care

Importantly, trauma outcomes result from complex interactions between patient factors, injury characteristics, and the care provided. As patient populations differ between level I and lower-level trauma centers, outcomes may also differ between these settings. To understand these interactions and interpret outcomes of care, all three types of variables should be considered.

### 3.1. Core Variables of Patient Characteristics, Injuries and Physiological Derangements

Patient characteristics and injury details form the basis of clinical outcomes. Core factors include age, sex, comorbidities, trauma mechanism, and injury severity. Injury severity should be described according to well established and internationally accepted coding, for instance by using a stated version of the Abbreviated Injury Scale (AIS) and the Injury Severity Score (ISS), with any threshold for severe injury predefined for the individual study [24,25,26]. These characteristics influence the patient’s physiological and immunological responses following trauma. Information on these systemic reactions completes the picture of the patient’s disease burden.

### 3.2. Care Process Variables of Indication, Timing and Extent of Interventional and Non-Interventional Care

The care process is closely related to patient outcomes. This includes the type, timing and intensity of interventions, that are guided by the patient’s clinical condition and may also interact with it. Non-operative aspects of care, such as medication use or ICU stay, are part of this process and may be associated with differences in complication risk. Capturing these process variables can help identify how care processes are related to patient outcomes and where care may be improved.

### 3.3. Outcomes Beyond Mortality

Outcomes should include mortality and length of hospital stay as well as complications and functional recovery. Mortality remains an essential and clinically robust outcome, but the primary endpoint should depend on the research question. Studies of rehabilitation or recovery may primarily assess functional outcomes, while studies of care processes may focus on complications or failure to rescue. Complications can link care processes to long-term outcomes such as functional recovery and quality of life. For example, the relationship between surgical timing, infection, hospitalization, and recovery should be evaluated with attention to injury severity and the indication for treatment. Complications therefore help describe how patient characteristics and care are related to final outcomes.

For each analysis, complications should be defined using explicit event criteria and graded using a generally accepted classification, such as the Clavien–Dindo classification [27,28], preferably with cases discussed, analyzed, and documented during weekly multidisciplinary morbidity and mortality meetings. Functional recovery should be measured using a named instrument, its scoring method, and a prespecified follow-up interval. The DNTR captures EQ-5D-based patient-reported outcome measures (PROMs), but, when used for scientific research, the version and timing must be specified [29]. Mortality and length of stay similarly require a defined observation period and consistent handling of transfers and readmissions.

## 4. Building the Data Infrastructure by Combining Registries

The institutional example described here combines three complementary sources within a major trauma center in the Netherlands. It illustrates how an integrated data infrastructure can support a Learning Healthcare System. It does not establish that a complete learning cycle is already operational. A complete cycle requires data collection and integration, analysis, feedback to clinical teams, implementation of a practice change, and reassessment of outcomes.

### 4.1. National Trauma Registry

**Figure 1:**
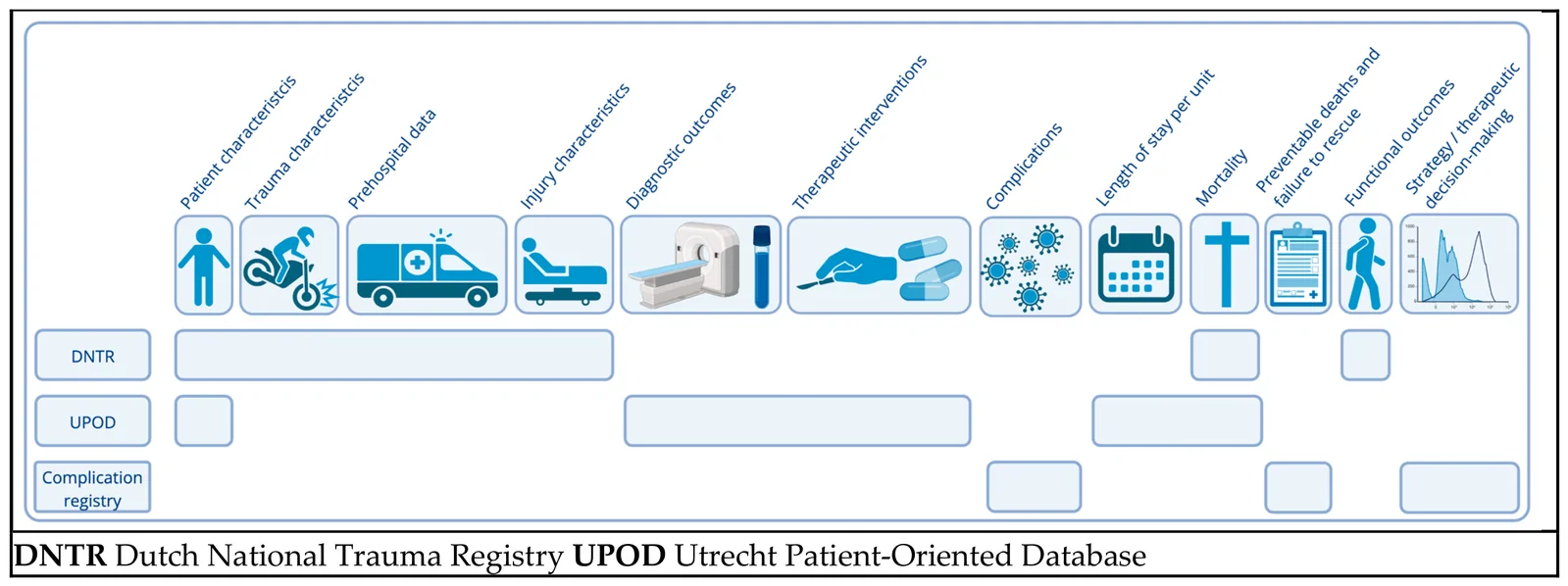
Data parameters and sources. The DNTR provides standardized patient and injury characteristics, prehospital data, mortality, and functional outcomes; UPOD provides detailed patient characteristics, diagnostic and therapeutic data, and length of stay; and the specialty complication registry provides clinician-recorded complications, preventable deaths and failure to rescue, and information on therapeutic decision-making. Overlap is resolved by selecting the preferred source for each variable as described in Section 4.4.

The DNTR includes patients with a traumatic injury who are admitted, transferred, or die within 48 h after Emergency Department (ED) presentation [29]. There are no exclusions based on age, injury type, admitting specialty, or length of stay [29]. The DNTR collects data based on the Utstein Template for Uniform Reporting of Data following Major Trauma and several additional parameters [30]. It includes patient, trauma, prehospital, and injury characteristics, mortality, and PROMs based on EQ-5D questionnaires [29] (Figure 1).

The Netherlands is organized into regional trauma networks [31]. Each region is responsible for robust retrospective data collection from all admitted patients after injury [31]. Reliable registration depends on structured documentation from clinicians and continuous quality checks. National participation and patient registration coverage have been reported as high [14], but these measures do not establish complete recording of every core variable or complete longitudinal follow-up. Patient-level coverage, variable-level completeness, and PROM response should therefore be reported separately and taken into account when building a combined learning system [10,11,23,32].

### 4.2. Utrecht Patient-Oriented Database

The Utrecht Patient-Oriented Database (UPOD) was developed to link laboratory, medication, and clinical data for research and innovation [33]. UPOD integrates data from multiple hospital information systems, including the electronic health record (EHR), laboratory information system, computerized physician order entry (CPOE) system, and a dedicated hematology database [33]. Data are periodically transferred into a central data environment, where they are structured and linked using a unique patient identifier and index date [33]. Because UPOD contains clinical data that can be repeatedly extracted, it supports repeated analyses of clinical practice, which can contribute to a Learning Healthcare System.

The custom-built traumatology database contains information on patient characteristics, diagnoses, therapeutic procedures, medication orders, blood product administration, laboratory results, and length of stay (Figure 1). Additionally, detailed hematology parameters generated by automated blood cell analyzers are stored for research purposes. Experienced data managers follow an ISO-9001 certified protocol to obtain the data [33]. Provided that clinical documentation is consistent, text-based searches can also be performed in the patient’s record, for example to identify recorded ATLS ABCDE findings.

The reliability of UPOD depends on continuous technical maintenance and specialized, dedicated and full-time personnel that operate in multidisciplinary teams to be able to extract routine care data in a meaningful way. Additionally, structured and uniform registration from clinicians is required [34,35]. Importantly, during data extraction from UPOD, clinical input and consultation between data managers and clinicians is required to ensure appropriate variable selection. For example, the choice between prehospital blood pressure measurements and the first ED blood pressure is dependent on the research question.

### 4.3. Specialty Complication Registry

In 2013 a complication registry was implemented in the Department of Trauma Surgery of the University Medical Center Utrecht, directly embedded in the EHR [3]. Physicians record adverse events for patients primarily treated by trauma surgeons, including events related to injury, human error, or logistics. Events are discussed weekly and classified using the National Trauma Data Standard (NTDS) and the Clavien–Dindo classification [27,28]. Furthermore, possible cases with failure to rescue or preventable deaths undergo in-depth analysis.

In contrast to the DNTR and UPOD, complications are primarily registered by clinicians (residents and physician assistants) alongside their regular clinical duties. This requires additional time, effort, institutional commitment and the willingness to record *all* adverse events. At this center, complication registration improved after responsibility was assigned to physician assistants with dedicated time and responsibility to cover the ward. Weekly multidisciplinary evaluation of adverse events provides direct feedback to clinicians and is an existing component of the learning cycle. Data extraction is supported by data managers through UPOD extraction from the EHR, but initial registration depends on clinical engagement.

### 4.4. Combination

Each registry captures a different (sometimes overlapping) part of the care pathway (Figure 1). Records are linked using the patient identifier numbers and index date [33].

The populations covered by the sources differ. The DNTR follows its trauma admission criteria, UPOD provides data available within the hospital information systems, and the specialty complication registry concerns patients primarily treated by trauma surgeons. The population eligible for a linked analysis therefore depends on the research question and overlap between sources.

Although several variables are available in more than one registry, the preferred source depends on how the information is recorded. The DNTR specifically captures patient characteristics, injury severity and injury characteristics by extracting information from the EHR and coding these variables according to internationally standardized classifications (such as AIS) [25]. While similar information can also be retrieved from UPOD, it is not recorded using these internationally standardized coding systems and may require extraction from multiple EHR fields or free-text documentation. Conversely, UPOD is the preferred source for routinely recorded physiological, laboratory variables and data on interventions, because these data are extracted automatically from structured fields within the EHR, minimizing the risk of manual transcription errors. The complication registry captures in-hospital adverse events scored by treating physicians, avoiding the need for further interpretation before data capture in the framework.

**Figure 2:**
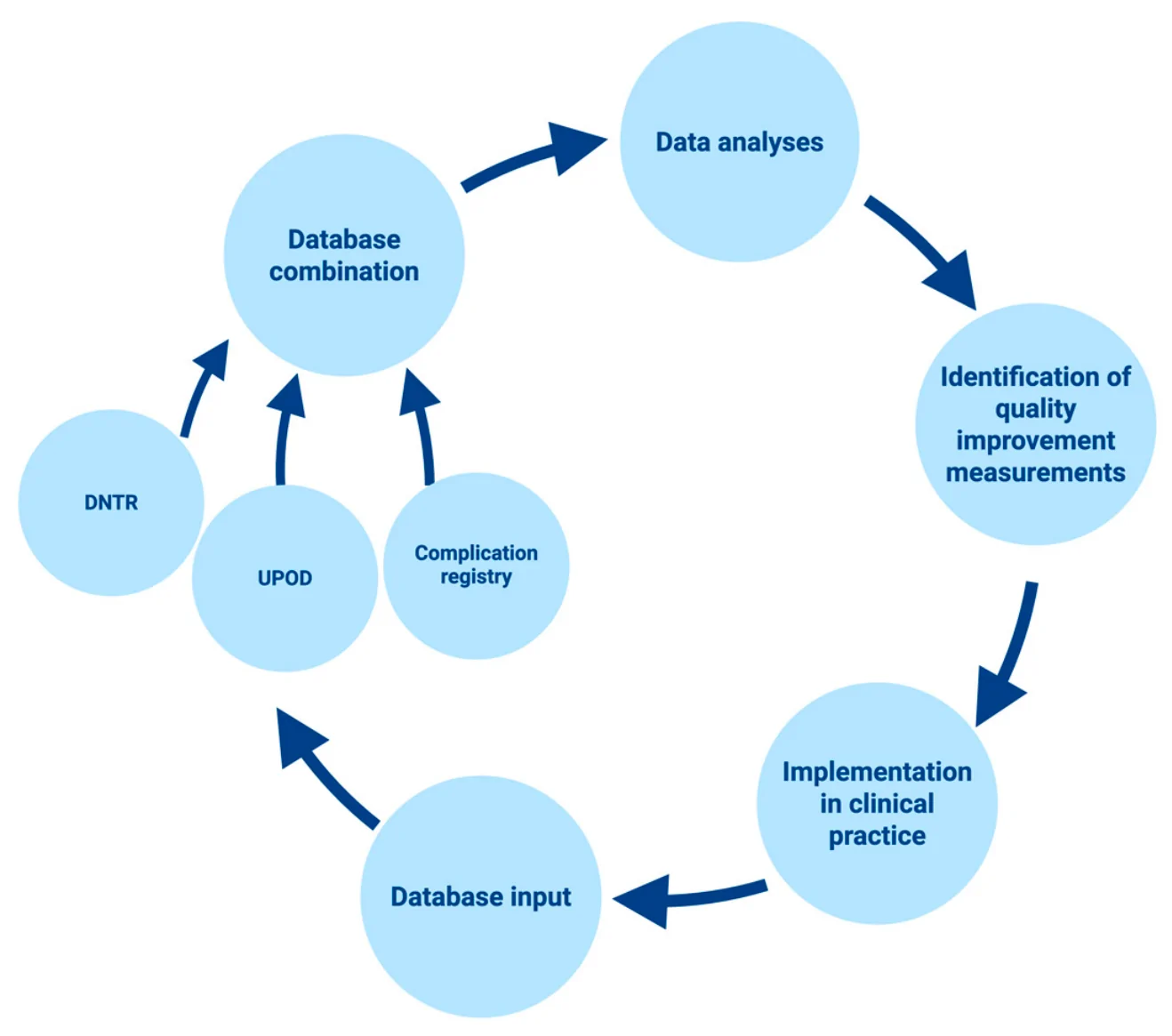
Proposed feedback loop from database integration to continuous quality improvement in clinical practice. Data from the UPOD, DNTR, and Complication Registry are integrated to provide the foundation for a Learning Healthcare System. The envisioned next steps involve analyzing these linked data to identify opportunities for quality improvement, implementing targeted interventions in clinical practice, and evaluating their effects through continued data collection. This proposed feedback loop illustrates the long-term objective of transforming the existing database infrastructure into a continuous cycle of data-driven learning and improvement in patient care.

Linking these systems through patient identifiers and injury events can create a comprehensive dataset (Figure 1). These linked registries provide the infrastructure for a Learning Healthcare System. However, integration of data sources alone does not constitute continuous improvement in care. Research enabled by the combined dataset includes a study of physiological and immunological parameters from UPOD in relation to injury severity from the DNTR and infectious complications from the complication registry [36]. Such research illustrates analytical use of linked data, rather than evidence of a complete feedback and improvement cycle (Figure 2). Completing the cycle requires collaboration between clinicians, researchers, data managers, and epidemiologists, as well as investment in personnel, data quality control, and institutional maintenance.

### 4.5. Ethics and Privacy

Secondary use of routine care data requires an appropriate ethical and governance framework, while unnecessary selection bias should be minimized [37,38]. Consent requirements and any alternative arrangements must be determined for each project under the applicable institutional framework. Pseudonymization, secure access, and transparent information about secondary use are important elements of data governance [37,38].

## 5. Clinical and Research Applications

The main advantage of an integrated data infrastructure lies in combining detailed data across the care pathway. This can support research on personalized care, prevention strategies, and the burden on society. The following applications describe opportunities enabled by the infrastructure; their clinical effectiveness is outside the scope of this paper.

### 5.1. Personalized Care

Detailed and integrated data can support the evaluation of more personalized treatment decisions and practice variation. Within a complete Learning Healthcare System, findings would be communicated to clinical teams, assessed for clinical relevance, and used to guide changes in care. The effect of those changes would then be reassessed. Whether the described infrastructure improves clinical decision-making or patient outcomes remains to be evaluated.

### 5.2. Prevention

Trauma registries help identify risk profiles based on patient characteristics and injury mechanisms. This enables the development of targeted prevention strategies and can inform public health policies, such as regulations on alcohol use [39,40].

### 5.3. Evaluation of Healthcare Systems

Trauma often leads to long-term disability, rehabilitation, and loss of productivity [7]. Quantifying this burden can support evaluation of healthcare system performance and help identify inefficiencies or vulnerable patient groups.

### 5.4. Methodological Considerations

Combining multiple data sources introduces methodological challenges when used for scientific research. Variable selection and statistical analyses should be guided by a predefined research question [41]. Confounders should be selected using prior literature and clinical expertise, rather than statistical criteria alone [41]. This paper presents a conceptual framework and does not analyze patient-level data, and therefore, a single statistical analysis plan or universal set of primary and secondary outcomes is not provided. The paper primarily describes a possible Learning Healthcare System build for continuous quality improvement of clinical practice. Each scientific study that uses this framework should specify its population, inclusion and exclusion criteria, index trauma episode, outcome definitions and time windows, primary and secondary endpoints, and analysis plan. Clinicians, researchers, epidemiologists, and data specialists contribute complementary expertise to this process [21].

## 6. Limitations and Transferability

The proposed learning cycle consists of collection and integration of routine care data, analysis of a defined clinical question, feedback to the relevant clinical team, agreed modification of practice, and reassessment using the same outcome definitions. In this institutional example, data integration and research use are described, and weekly complication review provides an existing feedback mechanism. Routine feedback based on the combined dataset and documented changes followed by outcome reassessment remain to be established or demonstrated. Future evaluations should record the question addressed, feedback process, practice change, implementation date, and subsequent outcomes.

This paper describes a single-center institutional implementation and does not evaluate the feasibility or effectiveness of the complete learning cycle. Coverage and completeness differ between the linked data sources and subsequently impact the possibility to analyze processes and quality control. For instance, high patient registration coverage does not imply complete recording of every variable or complication, and incomplete PROM follow-up may introduce selection bias. Complications are primarily recorded during hospital care, and events occurring after discharge or at other hospitals may be less well documented. Changes in registration responsibilities and practices may also affect complication ascertainment over time. Comparisons between periods should therefore distinguish changes in recording from changes in clinical outcomes.

The infrastructure depends on dedicated clinical engagement, structured clinical documentation, data managers, technical maintenance and multidisciplinary collaboration. Linkage errors, coding differences, and inconsistent definitions may further affect interpretation. Transferability depends on the availability of trauma registries, hospital data systems, dedicated personnel, and appropriate governance arrangements. Application in other trauma systems may require adaptation of the linkage procedures, outcome definitions, and feedback processes to local organizational and regulatory structures.

Future follow-up should evaluate linkage quality, variable completeness, timeliness, staff workload, implementation of feedback, and whether agreed changes are followed by measurable improvement in patient outcomes. Such evaluation is necessary to establish the practicality and clinical value of the proposed learning cycle over time and to determine its applicability across different trauma care settings.

## 7. Conclusions

Evaluation and improvement of trauma care should reflect the complexity of trauma care. Integrating patient and injury factors, care processes, and outcomes beyond mortality provides a more comprehensive description of clinical care. Linking existing registries offers a foundation for a Learning Healthcare System. Routine feedback from the combined dataset, subsequent practice changes, and reassessment are to be the result of a system such as the one described. Establishing and evaluating these steps is necessary to determine whether this infrastructure improves care for (severely) injured patients. Maintaining the infrastructure requires dedicated personnel, uniform clinical documentation, quality control, and sustained institutional investment.

## Data Availability

No new data were created or analyzed in this study. Data sharing is not applicable to this article. The manuscript describes existing clinical registries and data infrastructures but does not present or provide access to patient-level data. Access to data from these sources for future research is subject to the applicable institutional approval, governance, privacy, and data-access procedures.
